# Supplementary material for: In situ cryo-ET structure of phycobilisome–photosystem II supercomplex from red alga
Source: eLife. 2021 Sep 13;10:e69635. doi: 10.7554/eLife.69635 (PMC8437437; doi:10.7554/eLife.69635)
Supplement: Supplementary file 1. [file elife-69635-supp1.docx]

**Supplementary file 1. Cross-correlation coefficient (CCC) of crystal structures or single particle analysis model and sub-tomogram averaging map.**

| **Sub-tomogram averaging map** | **Fitted model** | **ccc** |
| --- | --- | --- |
| PBS1  (Double PBS-PSII) | SPA model (PDB code 6KGX) | 0.8846 |
| PBS2  (Double PBS-PSII) | SPA model (PDB code 6KGX) | 0.9049 |
| A PSII dimer  (Double PBS-PSII) | PSII crystal structure (PDB code 4YUU) | 0.9306 |
| B PSII dimer  (Double PBS-PSII) | PSII crystal structure (PDB code 4YUU) | 0.9495 |
| C PSII dimer  (Double PBS-PSII) | PSII crystal structure (PDB code 4YUU) | 0.9492 |
| A' PSII dimer  (Double PBS-PSII) | PSII crystal structure (PDB code 4YUU) | 0.9469 |
| B' PSII dimer  (Double PBS-PSII) | PSII crystal structure (PDB code 4YUU) | 0.9468 |
| C' PSII dimer  (Double PBS-PSII) | PSII crystal structure (PDB code 4YUU) | 0.8441 |
| C" PSII dimer  (Double PBS-PSII) | PSII crystal structure (PDB code 4YUU) | 0.8211 |
| PBS1  (PBS-PSII) | SPA model (PDB code 6KGX) | 0.7623 |
| PSII dimer A  (PBS-PSII) | PSII crystal structure (PDB code 4YUU) | 0.9962 |
| PSII dimer B  (PBS-PSII) | PSII crystal structure (PDB code 4YUU) | 0.9958 |
| Lateral hexamer  (PBS-PSII) | Hd (SPA model, PDB code 6KGX) | 0.820 |
| Lateral hexamer'  (PBS-PSII) | Hd (SPA model, PDB code 6KGX) | 0.8271 |
